# Considering the Lives of Microbes in Microbial Communities

**DOI:** 10.1128/mSystems.00155-17

**Published:** 2018-04-10

**Authors:** Elizabeth A. Shank

**Affiliations:** a Department of Biology, University of North Carolina, Chapel Hill, North Carolina, USA; b Department of Microbiology and Immunology, University of North Carolina, Chapel Hill, North Carolina, USA

**Keywords:** *Bacillus subtilis*, bacterial heterogeneity, cell-cell communication, microbial communities, microcosm, secondary metabolism, specialized metabolites

## Abstract

Over the last decades, sequencing technologies have transformed our ability to investigate the composition and functional capacity of microbial communities. Even so, critical questions remain about these complex systems that cannot be addressed by the bulk, community-averaged data typically provided by sequencing methods. In this Perspective, I propose that future advances in microbiome research will emerge from considering “the lives of microbes”: we need to create methods to explicitly interrogate how microbes exist and interact in native-setting-like microenvironments. This approach includes developing approaches that expose the phenotypic heterogeneity of microbes; exploring the effects of coculture cues on cellular differentiation and metabolite production; and designing visualization systems that capture features of native microbial environments while permitting the nondestructive observation of microbial interactions over space and time with single-cell resolution.

## Abstract (precis)

Over the last decades, sequencing technologies have transformed our ability to investigate the composition and functional capacity of microbial communities. Even so, critical questions remain about these complex systems that cannot be addressed by the bulk, community-averaged data typically provided by sequencing methods.

## PERSPECTIVE

This is an exciting time for those of us interested in microbial communities. The technologies available to us now (many hardly imaginable even a decade ago) are allowing new scientific questions to be asked and driving the field forward. For instance, sequencing technologies have provided us with fantastic insights into which microbes are present in specific environments as well as into what their transcriptional states are. However, as important as sequencing approaches remain, these methods typically provide only broad snapshots of the bulk behavior of microbial communities. Developing a deeper appreciation of the factors driving the establishment and stability of microbial communities, as well as a predictive framework to enable their rational perturbation, will require a better understanding of the causal relationships between microbial community members. As I illustrate here, a productive approach to do so is to consider “the lives of microbes.” In other words, we need to develop methods that capture the native features of the environments that these tiny organisms experience in the natural world and that allow us to explicitly interrogate how microbes exist and interact with one another at the appropriate spatial scale: the microscale.

## BACTERIAL TRANSCRIPTIONAL HETEROGENEITY

In an era of almost overwhelming sequencing data availability, it is easy to overlook the well-known fact that a single 16S rRNA gene sequence could represent multiple bacterial strains with widely variable genomic capacity. An even less appreciated fact, particularly with regard to microbiome data, is that even when we can determine the complete metagenomes present in a community, tremendous transcriptional heterogeneity exists at the level of individual bacterial cells, even within those with identical genotypes. This ability of bacteria to differentiate into transcriptionally and phenotypically distinct cell types has been well established in model microbes such as Bacillus subtilis (1), which we study in my laboratory. This cellular division of labor, or “bet-hedging,” among genetically identical sibling cells confers fitness advantages in some environments. A spate of recent reviews highlights the extent, origin, and benefits of phenotypic heterogeneity in bacterial populations (see, for example, references 2 and 3). Such cellular phenotypic heterogeneity is likely exhibited by all microbes, not only those as well studied as B. subtilis.

**FIG 1:**
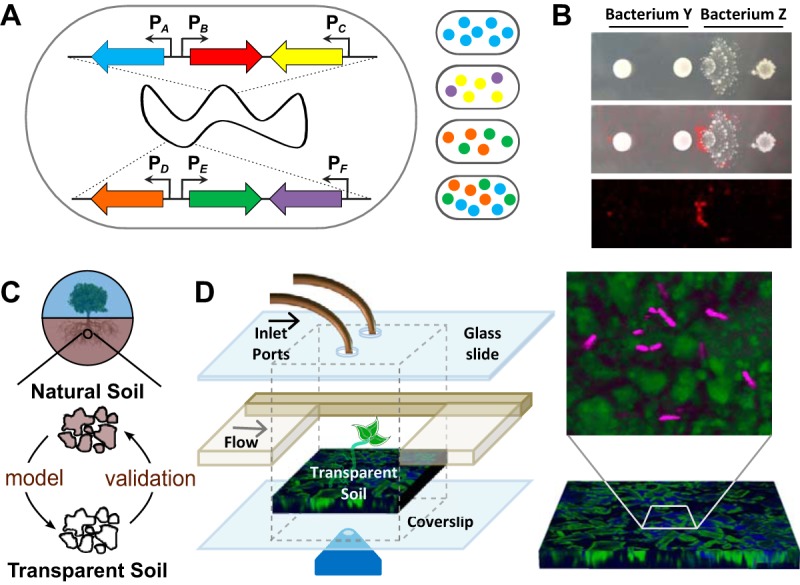
Schematic representations of methods that are revealing the “lives of microbes.” (A) Example of a strain containing modular fluorescent reporter constructs integrated into its genome at two different neutral loci. This combinatorial approach creates multiply fluorescent strains for use in investigating the relationships between different cells types using multispectrum imaging. "P_*X*_" indicates the promoter of gene *x* (e.g., a gene upregulated during bacterial differentiation or metabolite production); colored arrows represent genes encoding fluorescent proteins with distinct spectral properties. The right side of the panel illustrates four possible multiply fluorescent cells that could arise from this single genotype. (B) When grown in coculture, bacteria alter their phenotypes (as visualized by colony morphology; top panel) and metabolite production (as visualized by a false-color representation of a metabolite detected using imaging mass spectrometry; bottom panel). The center panel is an overlay image. (C) Synthetic study systems that capture some of the physical and chemical heterogeneity of natural soils will allow us to iteratively study microbial interactions in the laboratory and in their native environments. (D) We have developed microcosms containing a transparent soil-like substrate (green) that allows us to visualize individual microbes (magenta) and their cell-cell interactions in three dimensions over time.

The existence of bacterial transcriptional heterogeneity means that microbial communities consist of distinct microbial strains and that the individual cells within those populations are likely performing specialized tasks that cannot be inferred exclusively from sequencing data. Thus, to truly understand how microbes are interacting within communities, we need to understand not just which cell types each bacterial species can differentiate into but also where these specific cells are and who their neighbors are. My group is currently working on approaches to address both concerns. We are generating modular fluorescent protein constructs to detect multiple cell types simultaneously using spectral imaging (an approach equally applicable to other genetically tractable microbes) and are developing single-cell imaging systems to address the question of how cells physically partition themselves within microbial communities (Fig. 1).

## MICROBIAL METABOLITES AND COCULTURE INTERACTIONS

In their native environments, microbes most frequently live as members of complex multispecies communities. They are therefore surrounded not only by the neighboring cells that are physically present but also by the metabolic signals that these microbes have secreted. These secreted metabolites are primary drivers of microbial community interactions (4). More explicitly interrogating not just the transcriptional activity of individual cells but also the chemical signals that result from such gene activity will be critical to dissecting causal relationships within microbial communities; numerous such approaches are currently being pursued (5). Here I highlight two important aspects of how microbial metabolites are relevant to coculture interactions within microbial communities.

## EFFECTS ON BACTERIAL DIFFERENTIATION

For the last decade or so, Julian Davies (6) and others have argued that microbial metabolites naturally function as cell-cell communication signals rather than as killing agents. Even Waksman, who originally coined the term "antibiotics," noted in 1961 that “The existence of microbes that have the capacity to produce antibiotics in artificial culture cannot be interpreted as signifying that such phenomena are important in controlling microbial populations in nature” (7). Recent work in our laboratory supports this idea. Rather than taking the traditional approach of growing microbes in isolation, most of our research involves growing multiple bacterial species together. Using this approach, we have identified new biological roles (as interspecies chemical cues) for multiple metabolites that were previously ascribed only clinically relevant activities. Specifically, we recently demonstrated that thiocillin, an antibiotic produced by Bacillus cereus, has two structurally distinct activities: killing and biofilm induction (8). We further showed that even an antibiotic-null thiocillin variant retains its ability to activate biofilm gene expression. These data indicate that although we (with our human-centered focus on discovering clinically relevant drugs) have characterized thiocillin as an antibiotic, it is equally plausible that B. cereus instead evolved the ability to produce this metabolite for its biofilm-inducing properties. A similar argument could be made for numerous other microbial metabolites with multiple described bioactivities (9–12). New synthetic study systems (see below) should allow us to begin to address whether the clinically relevant or the cell-cell communication activities of these compounds (or both) are most pertinent in natural microbial communities.

## EFFECTS ON SPECIALIZED METABOLITE PRODUCTION

There are now substantial tantalizing data showing that novel metabolites are produced during mixed-species coculture (13). This is not surprising, given the substantial energy required by cells to generate the biosynthetic machinery needed to make these structurally complex metabolites. Since these coculture-specific metabolites are produced only in response to other microbes, they are promising candidates to be important cues mediating community interactions. My laboratory is therefore developing multiple workflows to uncover coculture-specific metabolites with interesting biological activities. We are also identifying whether there are genomic, phylogenetic, or biogeographic features that will allow us to effectively predict which cocultures are likely to yield coculture-specific metabolites. To do so, we are performing large-scale assays pairing hundreds of strains and then utilizing machine learning approaches to computationally determine whether particular metadata would allow us to predict high-value coculture combinations. Such efforts will accelerate our understanding of the interactions occurring within microbial communities by uncovering cell-cell communication cues, revealing which microbes might be interacting within previously characterized microbiomes, and guiding future discovery efforts.

## MICROCOSMS TO OBSERVE THE SPATIOTEMPORAL DYNAMICS OF MICROBIAL COMMUNITIES

In “thinking like a microbe,” we must remember that microbes typically exist at significantly lower densities and overall cell numbers than are usually examined in laboratory culture. Thus, we must establish methods to investigate populations of cells interacting at the spatial scale at which microbes natively exist: the microscale. Ideally, these study systems would capture some of the physical and chemical complexity of natural systems while still allowing the nondestructive visualization of the spatiotemporal dynamics of individual cells over time. Since my laboratory is focused on soil microbial communities, we are creating such native-setting-like microcosms using an optically transparent soil-like substrate (14); in some configurations, we can also incorporate plant roots (see Fig. 1). These optically transparent microcosms can be interrogated using confocal fluorescence microscopy; we have established that it is possible to image such microcosms over many weeks, opening the possibility of long-term observations of cell-cell interactions. In addition, we are developing these platforms to be amenable to Raman microspectroscopy. This method allows us to track the movement of nutrients through these communities using isotope labeling and should provide critical insights into their ecophysiology. Such studies, although not fully replicating natural systems, represent one step toward obtaining a more accurate perspective on the relevance of discoveries made on petri plates to the natural world. This work in my laboratory has fortuitously aligned with the goals of the EcoFAB initiative (see http://www.eco-fab.org/), which aims to promote a community of similarly minded researchers who are interested in creating controlled model ecosystems to study microbial communities in response to perturbations.

## FUTURE AREAS FOR DEVELOPMENT

In addition to the areas outlined above, technological advances in several areas would dramatically advance our understanding of microbial systems. Our ability to understand microbial interactions would be revolutionized by the ability to detect and identify “wild” and genetically intractable microbes, for instance, by using their distinct autofluorescence signatures. Although this would require significant advances in our optical detection capabilities, it would also substantially increase our ability to examine natural, nonmodel microbial systems. Other revolutionary advances will likely result from our improved ability to combine multiple imaging modalities (for instance, using methods that combine mass spectrometry with fluorescence and Raman microscopy) to simultaneously detect the spatiotemporal distributions of microbes and their metabolites at the microscale level within microcosms or natural environments. Such methods would allow us to dissect not only which cells are producing metabolites within microbial communities but also which microbes are altering their physiology and metabolism in response to these cues.
